# The influence of estimated retail tobacco sale price increase on smokers’ smoking habit in Jiangxi province, China: a cross-sectional study

**DOI:** 10.1186/s12971-015-0043-x

**Published:** 2015-08-19

**Authors:** Ruiping Wang, Liping Zhu, Wei Yan, Guang Zeng, Engelgau Michael

**Affiliations:** Songjiang Center for Disease Control and Prevention, 1050, North Xilin Road, Songjiang District, Shanghai, P.R China; China Field Epidemiology Training Program, Beijing, China; Jiangxi Provincial Center for Disease Control and Prevention, Nanchang, China; Center for Disease Control and Prevention, Atlanta, GA USA

**Keywords:** Smoking habit change, Tobacco retail price increase, Tobacco law legislation

## Abstract

**Introduction:**

China is the biggest tobacco producer and consumer in the world. Raising cigarette taxes and increasing tobacco retail prices have been prove as effective strategies to reduce tobacco consumption and the prevalence of smoking in western countries. But in China, it is uncertain how an increase of cigarette retail price will influence the tobacco consumption.

**Methods:**

From April to July, 2012, we selected 4025 residents over 15 years by a three stage random sampling in four cities, Jiangxi Province, China. We conducted interviews of their current smoking habits and how they would change their smoking behavior if tobacco retail prices increase.

**Results:**

Overall, the prevalence of smoking is 27 % (47 % for male, 3.1 % for female). 15 % of smokers have tried to quit smoking in the past but all relapsed (168/1088), and over 50 % of current smokers do not want to quit, The average cigarette price per pack is 1.1 USD (range = 0.25-5.0). If retail cigarette prices increases by 50 %, 45 % of smokers say they will smoke fewer cigarettes, 20 % will change to cheaper brands and 5 % will attempt to quit smoking. Smokers who have intention to quit smoking are more sensitive to retail cigarette price increase. With retail cigarette price increases, more smokers will attempt to quit smoking.

**Conclusion:**

Chinese smokers will change their smoking habits if tobacco retail prices increase. Consequently the Chinese government should enact tobacco laws which increase the retail cigarette price. The implementation of new tobacco laws could result in lowering the prevalence of smoking. Meanwhile, price increase measures need to apply to all cigarette brands to avoid smokers switching cigarettes to cheaper brands.

## Background

It is well known that tobacco use is a major health issue, and cigarette smoking continues to be the leading global cause of preventable death [27]. It kills nearly six million people and causes hundreds of billions of dollars of economic damage worldwide each year [11]. If current trends continue, by 2030 tobacco will kill more than 8 million people worldwide each year unless urgent action is taken.

China is the largest tobacco producer and consumer in the world, there are 300 million smokers in China, and 1million deaths are attributed to tobacco consumption each year [18]. The high prevalence of cigarette smoking and health hazard caused by tobacco use has aroused the attention of Chinese government. China signed the WHO Framework Convention on Tobacco Control (FCTC) in 2003, and implemented the FCTC in January of 2006. However a recently released official assessment report titled “*Tobacco Control and China’ Future*” shows that China has made limited progress toward tobacco control with poor performance in fulfilling its commitment to the FCTC [6, 29]. The major reasons including tobacco tax and price measures as well as warning sign on cigarette pack have not effectively implemented in China.

Raising the price of tobacco by taxation is an effective policy intervention for tobacco control which has been proved by many counties [14,16,19]. Although China introduced a cigarette tax increase in 2009, due to the fact that tobacco production is a state run enterprise that provides substantial revenues for the government, the tax changes have been absorbed at the government-owned producer level and have not affected the retail price (Gao *et al.*, 2012) (Global Adult Tobacco Survey Collaborative [7,8]). This indicates the open possibilities that remain in China for raising prices in an effective manner. In March 2011, the NPC (Nation People’s Congress) passed “China’s National Economic and Social Development, the 12th Five-Year Plan” which made it clear the outline of the next five years, to the full implementation of the banning smoking in public places through comprehensive measures such as tobacco control legislation, increase tobacco price and prohibit tobacco advertisement. All these embody the Chinese government’s determination on future tobacco control works.

In this paper, we mainly explore how smoking habits would be influenced if the tobacco retail price increases in Jiangxi province, which has not been reported before in China.

## Methods

### Study objects and sample size

This study was carried out from April to July 2012 in Jiangxi province located in the southeast of China. Jiangxi province had a middle ranking in forms of provincial GDP (Gross Domestic Product, GDP) with a population of 44.6 million and it encompassed 11 municipal cities in the year 2012. We took the Global Adult Tobacco Survey (GATS) as a reference to select study participants. The GATS is a nationally representative household survey of non-institutionalized men and women aged 15 years and older which employed a multistage sampling design. According to GATS 2010, the sample size is designed to produce estimates at the national level, by urban/rural classification, by gender and by the cross of gender and urban/rural with a 95 % margin of error of 3 % points or less for tobacco use rates of 40 %, assuming a design effect of 2.00 for estimates computed at the national level, a minimum expected respondent sample is 8000. In this study, we planned to interview 4100 residents which could be a representative sample at the provincial level. First, we randomly selected 4 cities (Jiujiang, Ganzhou, Shangrao and Yichun) from the 11 municipal cities, then five towns of each city were selected by a random number produced by computer, and then 205 households of each town were selected randomly, finally we selected only one person aged 15 or higher from each household of the 4100 households by using Kish Grid Sampling (KISH) code, totally we enrolled 4100 participants.

### Data collection and analysis

The Tobacco Questions for Surveys (TQS) are 20 key questions selected from GATS to track individual tobacco use behavior, attitudes toward smoking cessation and it contains questions that record individual spending on smoked tobacco products (questions including current tobacco smoking status, past smoking status, number of tobacco product smoked per day, tried to quit in past 12 months, the quantity and cost of last cigarette purchase, *etc.*). The current prevalence of smoking by demography were calculated by using participants’ responses, and smokers’ monthly spending on tobacco consumption were calculated by using the current tobacco retail price per pack multiplied by their monthly purchased packs, and the proportion of their monthly spending on tobacco purchase were calculated by using their monthly cost on tobacco consumption divided by their reported monthly income. Smokers’ quitting behavior was assessed by question of ‘During the past 12 months, have you tried to stop smoking?’ We also asked about smokers’ attitude toward smoking cessation (Do you have the intention to quit smoking? **A**. *Yes, I will quit smoking in a year*; **B.***Yes, I will quit smoking but not in a year*; **C.***No, I don’t want to quit*; **D.***I have never considered this issue*).

In this study we included three categories of smoking habit change: use fewer cigarettes, change to cheaper tobacco brands and quit smoking. Four additional questions were used to collect information on how smokers’ reported that their smoking habit would change with the hypothetical increase of tobacco retail price, and three categories of smoking habit change were derived from the four questions that following, all of which were used during face to face interviews. (Q1 ‘what’s tobacco retail price per pack you usually smoke? ***A indicates smoker’s answer to Q1***’, Q2 ‘if the tobacco retail price increase, to which price per pack you will consider smoking less? ***B indicates smoker’s answer to Q2***’, Q3 ‘if the tobacco retail price increase, to which price per pack you will consider to change a cheaper brand? ***C indicates smoker’s answer to Q3***’, Q4 ‘if the tobacco retail price increase, to which price per pack you will consider quitting smoking? ***D indicates smoker’s answer to Q4***’). We calculated the difference between the price that the smoker currently pays for tobacco (A) and the price that would influence the smoker to reduce tobacco consumption (B) to determine the price increase that would influence a smoker to smoke less (formula (B/A)). Similarly, we used the same process to calculate the price increase which would influence a smoker to change to a cheaper brand (formula (C/A)) and to quit smoking altogether (formula (D/A)).

Prices in TQS are originally recorded in Chinese local currency (RMB), we then converted them into US Dollar (USD) according to the exchange rate in May of 2012.

Data analyses were conducted with SAS 9.1. According to GATS sample weights manual, weights were computed for participants using reciprocals of inclusion probabilities. Departures from proportional allocation to cities were calibrated to the numbers of smokers in each age, sex, and education and occupation group. The prevalence of smoking was calculated by different demographic strata such as age, gender, and education as well as occupation; chi-square test was used to explore factors that influence smoker’s attitude toward smoking cessation and the attempt of quitting smoking. The relationship between change in smoking habit and the estimated increase of tobacco retail price was described by charts and Spearman correlation analysis was used to explore whether people who spend a higher proportion of their monthly income on cigarette purchase are more sensitive to price changes.

## Results

We finally interviewed 4025 participants with their informed consents which gave us a response rate of 98 %.

Among the 4025 participants, 54 % were male, of which 47 % were smokers. The age of participants ranged from 15 to 87 years old, with those aged 35–55 representing approximately half of the sample. The primary school or junior high school was the highest level of education achieved for 71 % of the sample, and only 5.4 % had received college education or above. In this survey, more than half of participants were peasants (farmers or fishermen), 31 % were workers (non-government employment) and the proportion of civil servants, students, and retired people combined was less than 20 %. We found that there was a statistically significant difference in the proportion of males and females who were smokers and non-smokers (*p* < 0.01), as well as with respect to age (*p* < 0.01) and occupation (*p* < 0.01) (Table 1).

**Table 1:** Demographic and prevalence of smoking among 4025 participants in Jiangxi, China, 2012

Demographic	Total number (% for total)	NO. of smokers	Prevalence of smoking (%)	*χ* ^*2*^	*p*	Prevalence of smoking by gender	
						Male (%)	Female (%)
**gender**				592	<0.01		
Male	2210 (54)	1030	47			-	-
Female	1815 (45)	58	3.2			-	-
**Age(years)**				36	<0.01		
15-24	385 (9.6)	36	9.4			20	0
25-34	492 (12)	132	27			50	0
35-44	1028 (26)	288	28			50	3.2
45-54	953 (22)	275	29			49	5.6
55-64	899 (24)	289	32			50	4.3
65-87	268 (2.7)	68	25			42	0
**Education**							
Illiterate	378 (9.4)	123	33	0.090	0.77	63	2.6
Primary school	1249 (31)	310	25			43	5.8
Junior high school	1600 (40)	437	27			48	1.4
Senior high school	580 (14)	153	26			41	2.2
College and above	218 (5.4)	65	30			47	0
**Occupation**				26	<0.01		
Peasant	2065 (51)	568	28			49	1.1
Worker	1233 (31)	416	34			51	6.6
Retired people	521 (13)	62	12			64	0
Civil servant	135 (3.4)	42	31			21	4.8
Student	71 (1.8)	0	0			0	0
Total	4025	1088	27			47	3.2

### Prevalence of smoking

In this study, 1088 out of 4025 participants (27 %) were smokers. The prevalence of smoking in males was 47 % (1030/2210) and 3.2 % in females (58/1815). The prevalence of smoking in the 6 age groups was relatively higher among male participants than in female participants (Table 1). Illiterate participants had the highest prevalence of smoking which was 63 % amongst males and 33 % among overall participants, and the prevalence of smoking in the other four education categories ranged from 25 % to 30 % (Table 1). The prevalence of smoking in the five occupational categories was over 27 % except for retired and students (Table 1).

### Smoking cessation behavior and attitude

Among the 1088 smokers, 168 smokers (15 %) tried quitting smoking in the past, including 165 males and three females, of which 16 % male smokers and 5.2 % female smokers tried quitting smoking, and the quitting smoking attempt rate between male smokers and female smokers was statistically significant (*χ*^*2*^ = 4.95, *p* < 0.05), but all of the 168 smokers relapsed. When asked about their attitudes toward smoking cessation, over 50 % of smokers reported no intention to quit smoking, 31 % of smokers reported they have never consider this issue before, and only 19 % of smokers reported intention to quit smoking, of which 6.7 % indicated interest in quitting within a year.

### Tobacco retail price and smoking cost

In this study, the average tobacco retail price per pack that smokers usually smoked was 1.3 USD (range 0.28 USD to 5.5 USD per pack), the estimated tobacco retail price per pack that induce less smoking was 1.8 USD (Standard Deviation, *SD* = 1.3 USD), the estimated tobacco retail price per pack that induce switching to cheaper brands was 2.1 USD (*SD* = 1.4 USD) and the estimated tobacco price per pack that lead to quit smoking was 3.4 USD with a SD of 2.6 USD. 63 % of smokers usually smoked low price tobacco brands (range 0.28 USD to 1.7 USD per pack), 32 % of smokers smoked medium price tobacco brands (range 1.8 USD to 2.4 USD per pack), and only 4.9 % of smokers chose the relatively expensive tobacco brands (range 2.5 USD to 5.5 USD per pack). The amount of money that smokers in Jiangxi province spent on tobacco each month ranged from 4 USD to 135 USD with an average of 35 USD which accounted for 15 % of their monthly income.

More than 60 % of smokers thought the tobacco retail price was reasonable or cheap. Meanwhile, 34 % of smokers thought the cigarette retail price was extremely expensive and about 5.3 % of smokers thought the cigarette retail price was expensive. Table 2 indicates that smoker’s attitude to tobacco retail price was related to their current views on smoking cessation. Smokers who thought the tobacco price was extremely expensive or expensive were more likely to consider quitting smoking (the proportion was 28 %, 119/432), than smokers who thought the tobacco price was reasonable or cheap (the proportion was 14 %, 90/656), (*χ*^*2*^ = 26, *p* < 0.05).

**Table 2:** The influence of smokers’ attitude toward tobacco retail prices on smoking cessation attitude among smokers in Jiangxi, China, 2012

Smokers’ attitude toward tobacco retail price	Smoking cessation attitude (NO. of smokers, (%))				Total
	Quit in a year	Quit but not in a year	Not quit	Never thought about it	
Extremely expensive	40 (11)	56 (15)	152 (41)	126 (34)	374
Expensive	6 (10)	17 (30)	23 (40)	12 (20)	58
Reasonable	26 (4.4)	64 (11)	365 (63)	127 (22)	582
cheap	0 (0.0)	0 (0.00)	26 (35)	48 (65)	74
**Total**	**72**	**137**	**566**	**313**	**1088**

### Smoking habit change with estimated tobacco retail price increase

**Fig. 1:**
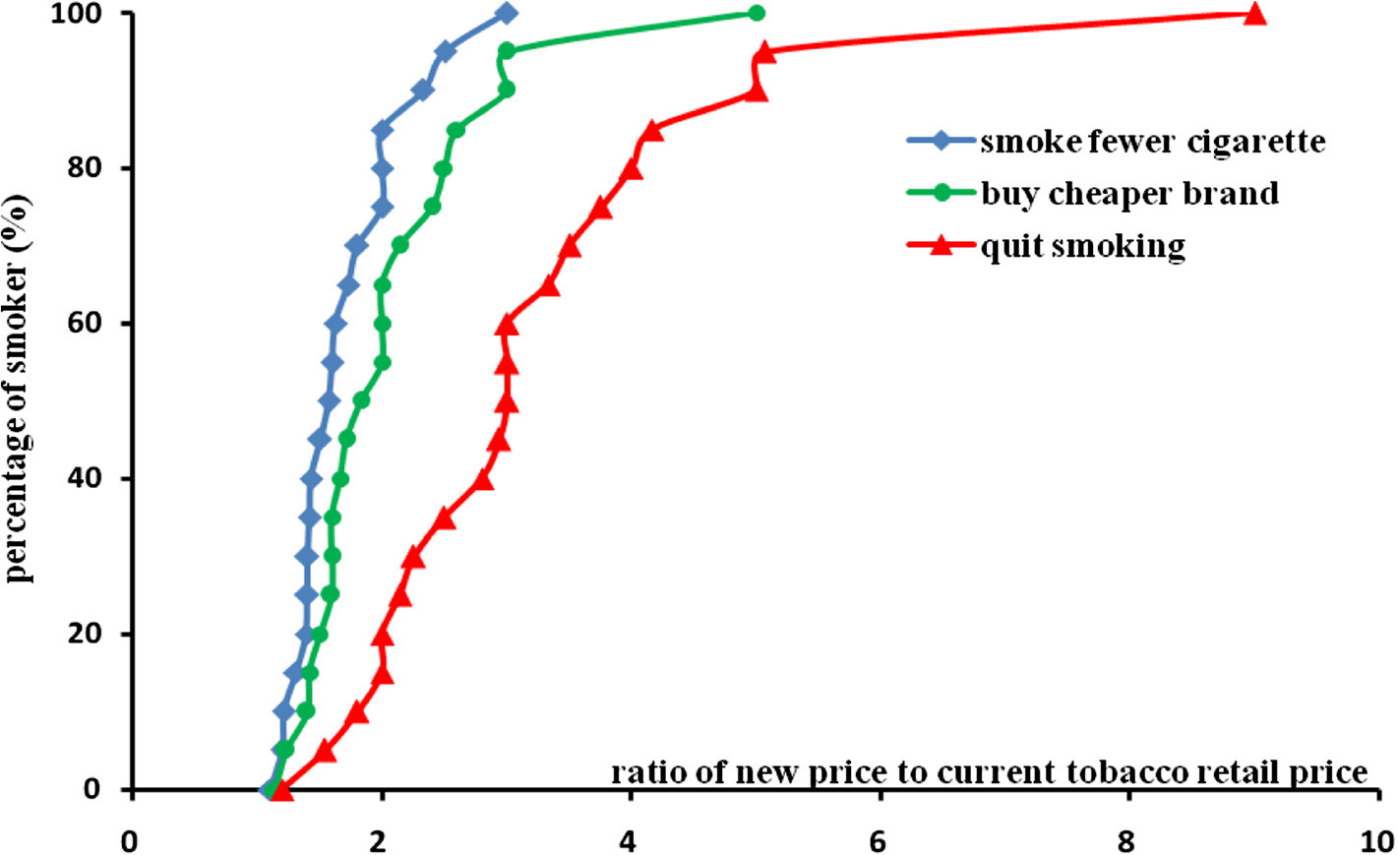
The percentage of smokers who will change smoking behavior with the increase of tobacco retail price in Jiangxi, China, 2012. Note: Y axis indicates the percentage of smokers who will change their smoking behavior, X axis indicates the ration of a new increased estimated tobacco retail price to the current tobacco retail price, 1 = current price, 2 = twice current price (double), 3 = three times the current price (triple), the same as the following

Figure 1 shows the relationship between smoking habit change and the estimated increase of tobacco retail price. When smoking habit change was arranged into three categories (smoke fewer cigarettes, smoke cheaper brands and quit smoking), we found that quit smoking required the largest increase in tobacco retail price. We found that as tobacco retail price increased, most smokers chose to smoke fewer cigarettes or to change to a cheaper brands rather than consider quitting smoking unless the tobacco retail price rose to very high levels (Fig. 1) which meant that lowest price needed to induce decreased smoking or cheaper brand switching was less than that need to induce motivation to quit.

We could use these data to estimate the proportion of smokers who will change their smoking habit (smoke fewer cigarette, smoke cheaper brand or quit smoking) if the tobacco retail price increases. For instance, if the tobacco retail price doubled, 84 % of smokers might choose to smoke fewer cigarettes, 62 % of smokers might chose to smoke cheaper brands, and only 21 % of smoker would consider quitting smoking.

**Fig. 2:**
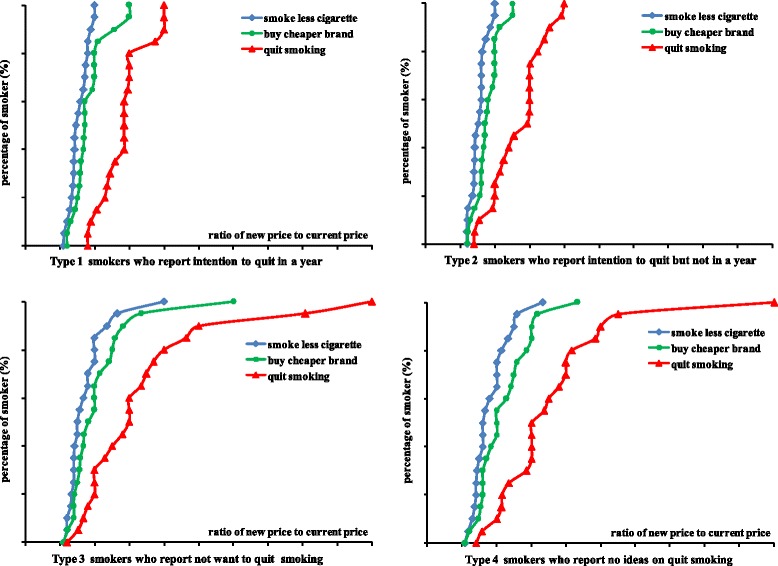
Four types of smokers who change smoking habit with the increase of tobacco retail price in Jiangxi province, China, 2012. Note: Y axis indicates the percentage of smokers who will change their smoking behavior, X axis indicates the ration of a new increased estimated tobacco retail price to the current tobacco retail price, 1 = current price, 2 = twice current price (double), 3 = three times the current price (triple), the same as the following

This study also showed that smokers who reported intention to quit smoking in the future were more sensitive to price increases, therefore they were more likely to quit if the tobacco price increased in comparison with smokers who had no intention to quit smoking or who had no ideas on smoking cessation (Fig. 2).

**Fig. 3:**
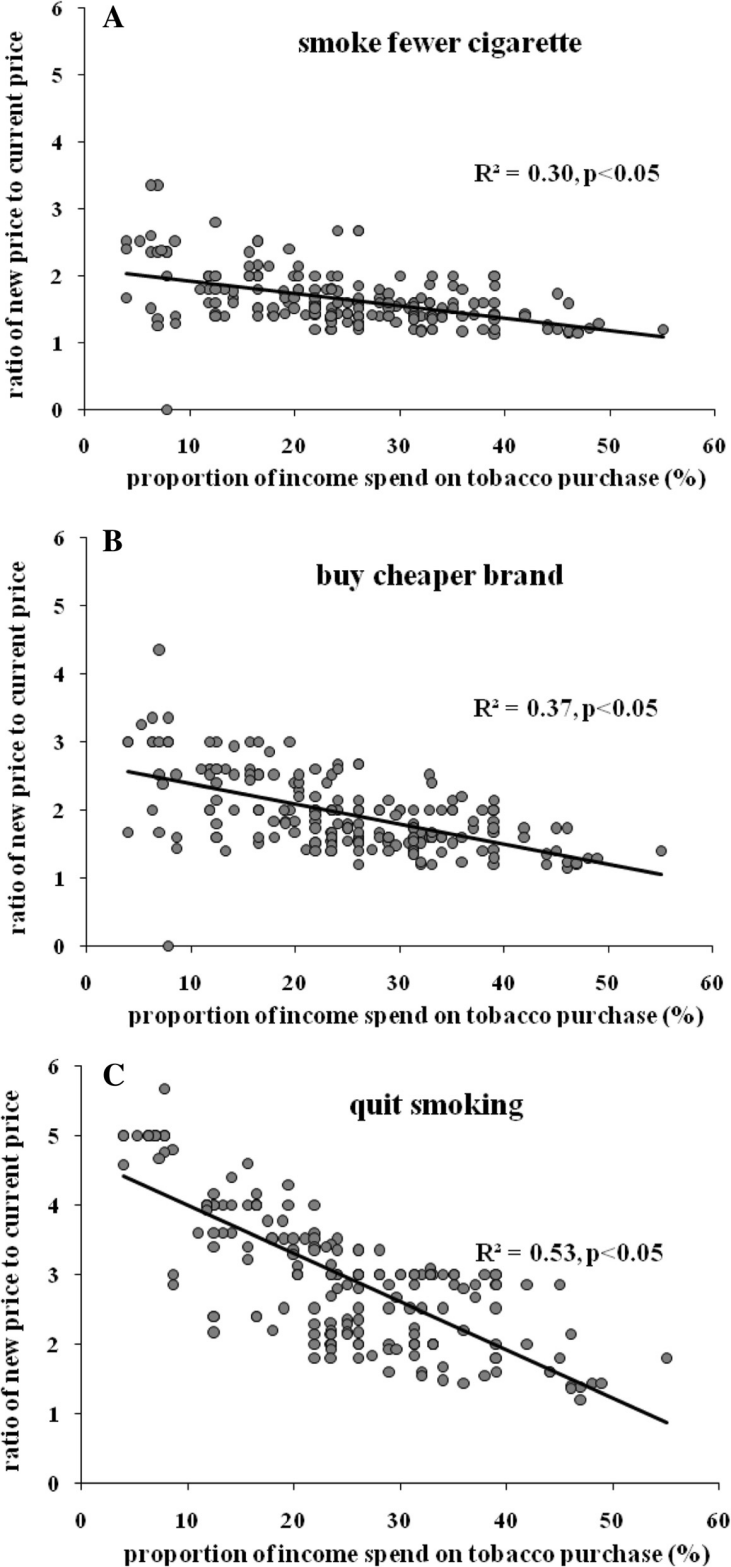
The correlation between the proportion of smokers’ income spend on tobacco purchase and their smoking habit change with the tobacco retail price increase, Jiangxi, China, 2012. Note: X axis indicates the proportion of smokers’ income spend on tobacco purchase, Y axis indicates the ration of a new increased tobacco retail price to the current tobacco retail price, 1 = current price, 2 = twice current price (double), 3 = three times the current price (triple), the same as the following. Chart A represents smokers who would smoke fewer cigarette if tobacco retail price increased, chart B represents smokers who would buy cheaper cigarette brand if tobacco retail price increased, and chart C represent smokers who would consider quitting smoking if tobacco retail price increased

Spearman correlation analysis results have been shown that people who spend a higher proportion of their monthly income on cigarette purchase are more sensitive to tobacco retail price changes. The spearman correlation coefficient was −0.54 (r^2^ = 0.30, *p* < 0.05) for smoke fewer cigarette, −0.61 (r^2^ = 0.37, *p* < 0.05) for buy cheaper cigarette brand, and −0.73 (r^2^ = 0.53, *p* < 0.05) for quit smoking (Fig. 3).

## Discussion and conclusion

For decades, tobacco marketing has been used to portray tobacco use in a favorable light, normalize smoking, underplay the associated heath risks and ultimately undermine tobacco control efforts [2,24,26]. But the lethal hazards induced by tobacco use have been proven by many researches and investigations since the 1920’s [3]. The WHO Framework Convention on Tobacco Control currently has 172 signed countries covering almost 90 % of the global population, and obligates member parties to introduce comprehensive bans on tobacco marketing to cut down tobacco consumption and finally lessen tobacco hazards [25].

In recent years, many countries around the world have taken the initiative to control cigarette consumption because of its impact on public health and healthcare cost [11]. China is in a unique position because its high smoking prevalence and the government manipulated tobacco industry provides a large tax base and abundant revenue (Gao *et al.*, 2012), but the unique position also makes it tough to implement tobacco control work in this country, although China had signed the FCTC and declared its commitment.

In this study, we found that the prevalence of smoking among Jiangxi residents was 27 %, with the prevalence of 47 % for males and 3.2 % for females respectively. In comparison with the Chinese national survey [4] in 1996 (63 % of adult male and 3.8 % of adult female were current smokers) and the Chinese risk behavior surveillance [28] in 2012 (66 % of adult male and 3.1 % of adult female were current smokers), the prevalence of smoking in Jiangxi province was relatively lower. But subsequent analysis has been shown that the prevalence of smoking among different age groups was all very high, this high prevalence of smoking in all age groups might lie in the ‘Chinese specific cigarette culture’. In China, cigarette sharing was a common phenomenon. Some people use cigarettes to initiate a conversation, some people view cigarettes as an ideal present for elders, friends and leaders, and cigarettes were commonly used during social gatherings such as wedding ceremonies, new-born baby celebrations, funeral ceremonies and all sorts of banquets, *etc.* This special cigarette culture combined with the low cigarette retail price and lack of purchase limitation lead to the high prevalence of smoking in all age groups, and people of different occupations and educational backgrounds.

Moreover, more than 50 % of smokers reported that they had no intention to quit smoking, and 31 % of smokers had never considered the smoking cessation issue. In accordance with the stage change model of health promotion theory [20], smokers in Jiangxi province were mostly in the first two primary stages (stage of no plan to change, stage of plan to change), this fact suggested that smokers in Jiangxi province generally had poor recognition on the hazards of tobacco use and had lower intention to change their smoking habit, so there was still a long way to go for tobacco control in China.

Tobacco use induced substantial social, economic and health costs have lead many countries to adopt higher cigarette taxes as a policy to reduce smoking [22]. The WHO’s FCTC has called for higher taxes and prices for tobacco products, and WHO has included raising taxes as a primary component in its MPOWER strategies (***M***onitor tobacco use and prevention policies, ***P***rotect people from tobacco smoke, ***O***ffer help to quit tobacco use, ***W***arn about the dangers of tobacco, ***E***nforce bans on tobacco advertising, promotion, and sponsorship, ***R***aise taxes on tobacco, ***MPOWER***) for tobacco control [7,8]. China introduced a cigarette tax increase in 2009, but promulgating a tax rate increase was not sufficient to reach meaningful tobacco control in China, the 2009 tobacco tax adjustment provides an example of what happens when a tobacco monopoly company operating under government management pursues its political or social objectives that do not include an increase in the retail price (Gao *et al.*, 2012) (Global Adult Tobacco Survey Collaborative Groups [7,8]). This is a critical reason the prevalence of smoking in China is still very high, and also misses the opportunity to see how the increase in tobacco tax and price will influence smoker’s habits. In this survey, by investigating smokers’ intention on smoking habit change with the estimated increase of tobacco retail price, we attempt to estimate the relationship between smoking habit change and tobacco retail price increase which could provide baseline information for the draft of tobacco control laws in China.

Lines of smoking behavior change in Fig. 1 can be used to estimate the proportion of smokers who will change their smoking habits if the tobacco retail price is increased to a new price. Literature mostly agrees that cigarette taxes and tobacco price increases are in general effective [5], and the effectiveness of cigarette taxes depends on how smokers view on such tax and price increase [23] [21]. This study shows that smokers will change their smoking habits with the increase of tobacco retail price, combined with the findings that smoker’ attitudes toward tobacco retail price is related with their view on smoking cessation, we can deduce that with the increase of tobacco retail price, smokers cannot bear the expensive tobacco price, so increased number of smokers will consider changing their smoking habit and even quit smoking, and their intention to change smoking habit can in turn makes them more sensitive to tobacco retail price increase, this is a positive impetus which may make more smokers quit smoking with the increase of tobacco retail price. An empirical regularity is that cigarette demand is relatively more elastic for low income smokers than high income smokers (Gospodinov and Irvine [9]), Gruber show that individuals in the lowest income quartiles are most sensitive to cigarette prices and those in higher quartiles are least sensitive (Gruber and Koszegi [10]). In this study, smokers who spend a higher proportion of their monthly income on cigarette purchase are more sensitive to tobacco retail price changes, which was consistent with previous study results.

A feature of cigarette market in China is the considerable variability of prices across brands (Hu *et al.* [12]), and the range in prices per pack in Chinese stores routinely vary 10-fold and in some stores 50-fold or more, this wide price spread across brands makes it easy for smokers to switch to cheaper brands in China (Justin *et al.* [15]). So we should notice that the wide range of tobacco retail price in Jiangxi China makes the tobacco control work through price increase much more complicated, this is because the large tobacco retail price difference provides opportunity for substitution from higher priced to cheaper products. Huang estimate a price elasticity of consumption of −0.13 between 2006 and 2009 (Huang *et al.* [13]),and Mao use national data to estimate a price elasticity of −0.15 (Mao *et al.* [17]), the overall lack of price sensitivity in China raises the public health concern that tobacco tax policy will have little impact on smoking behavior. So we recommend the tobacco retail price increase measures need to apply all cigarette brands to avoid smokers switching to cheaper brands. With the rising incomes among residents in China, the tobacco products are relatively more affordable (Blecher and Van [1]), so it’s also critical to increase tobacco retail price by a larger times to offset the increase of tobacco affordability and discourage consumption, as well as induce more smokers to quit.

This study is the first attempt to estimate how smoker’s smoking habits is influenced by the hypothetical tobacco retail price increase in China. Four thousand one hundred residents were selected from four out of 11 municipal cities in Jiangxi province by a three stage sampling combination with the application of KISH code, the sample size in this study was over half of the GATES recommended national level which could be a preferable estimation of the provincial level, although bias might be induced by the selection of four municipal cities to represent the whole province. Other limitations in this study include the information collected in this survey is attitudes of smokers which may not be a good representative of actual behavior change, and there is still lack of tobacco price increase measure in China which impede the observation of the real influence of tobacco retail price increase on smoker’s smoking habit change, possible another limitation in this investigation.

Areas for future research including carrying out a similar investigation at the national level to provide broader and more detailed information for the legislation of tobacco control laws, and tracing smokers’ real behavior change with the tobacco price increase when price measures be implemented in the near future, and exploring the influence of participants’ tobacco use knowledge on behavior change to identify whether poor knowledge can explain the observed low motivation to quit and so on.

### Ethical approval

Jiangxi Provincial Center for Disease Control and Prevention Ethics Board
